# Diagnostic prediction models to identify patients at risk for healthcare-facility–onset *Clostridioides difficile*: A systematic review of methodology and reporting

**DOI:** 10.1017/ice.2023.185

**Published:** 2024-02

**Authors:** William M. Patterson, Jesse Fajnzylber, Neil Nero, Adrian V. Hernandez, Abhishek Deshpande

**Affiliations:** 1 Cleveland Clinic Lerner College of Medicine of Case Western Reserve University, Cleveland, Ohio, United States; 2 Health Outcomes, Policy, and Evidence Synthesis (HOPES) Group, University of Connecticut School of Pharmacy, Storrs, Connecticut, United States; 3 Unidad de Revisiones Sistemáticas y Meta-análisis (URSIGET), Vicerrectorado de Investigación, Universidad San Ignacio de Loyola (USIL), Lima, Peru; 4 Education Institute, Floyd D. Loop Alumni Library, Cleveland Clinic, Cleveland, Ohio, United States; 5 Center for Value-Based Care Research, Primary Care Institute, Cleveland Clinic, Cleveland, Ohio, United States; 6 Department of Infectious Diseases, Respiratory Institute, Cleveland Clinic, Cleveland, Ohio, United States

## Abstract

**Objective:**

To systematically review the methodology, performance, and generalizability of diagnostic models for predicting the risk of healthcare-facility–onset (HO) *Clostridioides difficile* infection (CDI) in adult hospital inpatients (aged ≥18 years).

**Background:**

CDI is the most common cause of healthcare-associated diarrhea. Prediction models that identify inpatients at risk of HO-CDI have been published; however, the quality and utility of these models remain uncertain.

**Methods:**

Two independent reviewers evaluated articles describing the development and/or validation of multivariable HO-CDI diagnostic models in an inpatient setting. All publication dates, languages, and study designs were considered. Model details (eg, sample size and source, outcome, and performance) were extracted from the selected studies based on the CHARMS checklist. The risk of bias was further assessed using PROBAST.

**Results:**

Of the 3,030 records evaluated, 11 were eligible for final analysis, which described 12 diagnostic models. Most studies clearly identified the predictors and outcomes but did not report how missing data were handled. The most frequent predictors across all models were advanced age, receipt of high-risk antibiotics, history of hospitalization, and history of CDI. All studies reported the area under the receiver operating characteristic curve (AUROC) as a measure of discriminatory ability. However, only 3 studies reported the model calibration results, and only 2 studies were externally validated. All of the studies had a high risk of bias.

**Conclusion:**

The studies varied in their ability to predict the risk of HO-CDI. Future models will benefit from the validation on a prospective external cohort to maximize external validity.

*Clostridioides difficile* (formerly *Clostridium difficile*) is the most common cause of healthcare-associated diarrhea in the United States and is associated with increased morbidity, mortality, and a mean attributable cost of US$21,448.^1,2^ Elderly individuals, those receiving antibiotics, and those with a prolonged hospital stay are at an increased risk of healthcare-facility–onset (HO) *C. difficile* infection (CDI).^3^ Numerous studies have developed diagnostic risk-prediction models to identify inpatients with the greatest risk of developing HO-CDI. Two potential advantages of these risk-prediction models are that they can help clinicians intervene by (1) optimizing patients’ exposure to high-risk antibiotics during their hospitalization and (2) targeting them for preventive treatment. However, the accuracy, quality, and clinical utility of these prediction models remain uncertain, which may contribute to their limited adoption in clinical settings.

Prediction models can be categorized as diagnostic or prognostic. Diagnostic models predict the occurrence of a disease state (eg, development of HO-CDI), whereas prognostic models predict outcomes within a specific disease state (eg, recurrence and mortality in patients with CDI).^4^ In the field of infectious diseases, both types of models are commonly used to guide clinical decision making (eg, identifying individuals who would benefit the most from Paxlovid). However, risk models for CDI have not been widely adopted.^5^ Understanding the reasons for limited uptake can aid future researchers in model design and reporting. A review of all clinical prediction models published in 6 high-impact journals in 2008 found significant issues, such as unclear study designs, lack of prospective validation cohorts, dichotomization of continuous variables, clear overfitting, or no calibration.^6^ The objective of our study was to systematically review and critically assess the methodology, reporting and generalizability of current diagnostic risk prediction models for HO-CDI in adults.

## Methods

The study procedures, methods, and reporting adhere to the 2020 Preferred Reporting Items for Systematic Reviews and Meta-Analyses (PRISMA) statement and TRIPOD-SMRA guidelines.^7,8^ The guideline checklist is available in the Supplementary Material (online).

### Study selection

Eligible articles included those presenting new multivariable diagnostic models, evaluating prior models with new data for predicting the risk of developing HO-CDI, and extending previously published models (considered a new model). We defined ‘diagnostic model’ as any statistical model predicting the risk or odds of inpatients developing HO-CDI ≥48 hours after admission.^9^ We included studies of any design, language, and publication date before January 21, 2022, with any timeframe (eg, prospective, retrospective). We excluded studies that included pediatric patients (aged <18 years), community-associated CDI, performed solely univariate analysis, used control group of patients with diarrhea, or described diagnostic models for individuals with a diagnosis of CDI.

### Search strategy

We conducted simultaneous searches of Medline (Ovid), EMBASE (Ovid), and the Cochrane Library from inception until January 20, 2022. A comprehensive search strategy was developed with a medical librarian (N.N.) using the following terms: (*Clostridioides* OR *Clostridium* OR *C difficile*) AND (predict* OR risk*) AND (model* OR tool*). Indexing terms and keywords were combined with Boolean and proximity operators. No search limits were applied, and an additional 18 records were identified by manually searching a narrative review. The complete search strategy for all queried databases is available in the Supplementary Material (online).

The article selection process occurred in 2 rounds using the Covidence systematic review software (Veritas Health Innovation, Melbourne, Australia). Records with abstract-only content (conference proceedings) were excluded due to insufficient content to analyze the model methodology. Two independent evaluators (W.P. and J.F.) screened and assessed search results, resolving discrepancies through joint review and consultation with an independent third researcher (A.D.). Full-text of all titles that met the inclusion criteria by a majority vote were obtained and evaluated to make a final decision to include or exclude the article from the study. In all the rounds, reviewers referenced the systematic review question to determine study inclusion. Articles selected for review were evaluated using prespecified model evaluation criteria informed by a literature review and expert opinion, in addition to the PRISMA guidelines.^7^

### Evaluation of development and validation of diagnostic models

Data were independently extracted (W.P. and J.F.) using the checklist for critical appraisal and the data extraction for systematic reviews of prediction modelling studies (CHARMS) checklist.^10^ Discrepancies were resolved through discussion and consultation with a third author (A.D.). Extracted data included sources, countries, study populations, participant types (eg, ICU, medicine ward), outcome to be predicted, candidate predictors, number of events, sample sizes, missing data, model development, performance, evaluation, data presentation, interpretation, and discussion.

To evaluate the risk prediction models, we defined a priori the criteria to determine a successful prediction model: (1) clearly state both target population and the outcome of interest; (2) ensure there is a representative sample with adequate size both for model development and validation; (3) use statistical methods appropriate for the question they are asking (eg, decision trees, logistic regression) and use cross-validation, bootstrapping (random sampling with replacement), and independent validation sets for internal and external validity; and (4) evaluate their discrimination and calibration, using measures such as the area under the receiver operating characteristic (ROC) curve and a calibration plot or table comparing predicted and observed outcome probabilities. We also assessed model performance using sensitivity, specificity, positive predictive value (PPV), negative predictive value (NPV), and positive/negative likelihood ratios (LR+/LR−).

### Risk of bias and applicability assessment

Two reviewers (W.P. and J.F.) independently assessed each study for risk of bias (ROB) using PROBAST (Prediction model Risk Of Bias ASsessment Tool).^11^ ROB refers to the potential bias introduced by the study design, conduct, or analysis on the predictive performance of the model. PROBAST provides questions that assess ROB in four domains (participants, predictors, outcome, and analysis) and concerns regarding applicability in 3 domains of prediction models: high, low, or uncertain. Concerns for applicability occur when study elements do not align with the review question (ie, studies conducted in large academic medical centers in major cities might not be as applicable to rural settings).

### Statistical analyses

Categorical factors were summarized as frequencies and percentages, whereas continuous measures were described as means, standard deviations (SD), medians, and interquartile ranges (IQRs). We calculated the relationship between the number of events in a sample set, the number of model candidate coefficients (events per variable or EPV), and a correlation coefficient. An EPV >10 is generally accepted to maintain bias and variability at acceptable levels.^12^ No meta-analysis (quantitative synthesis of the data) was conducted. All analyses were performed using R version 4.1.0 statistical software (R Core Team, Vienna, Austria).

## Results

### Study selection

**Figure 1.**
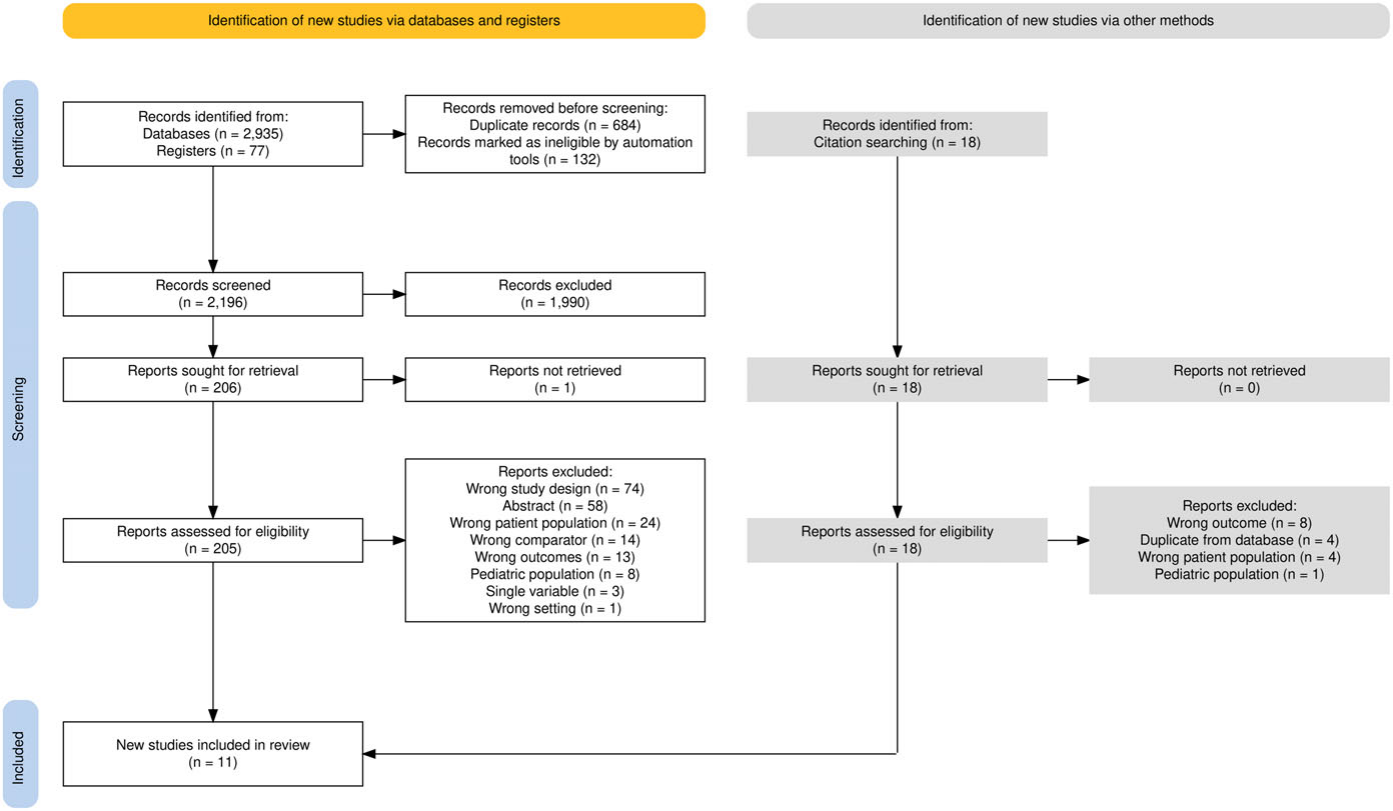
PRISMA flowchart of study selection.

After full-text review, 11 citations were eligible (Fig. 1). Two groups developed an index model to be validated on external data in a separate, later study (one of these proposed an additional, updated model).^13–16^ Two studies reported 2 risk-prediction models each; thus, our review assessed a total of 12 risk-prediction models from 11 articles.^17,18^ We used 11 as the denominator when reference was made to studies and 12 when reference was made to prediction models.

### Study details

In total, 10 (91%) studies were conducted in North America and 1 (9%) in Europe. Also, 6 studies (55%) were conducted at a single-center healthcare facility, and 5 (45%) studies were multicenter studies ranging from 2 to 6 centers. No other international studies were included.

### Data sources

All studies used retrospective data from electronic medical records for model development. Three models (25%) were validated with bootstrapped simulations (Table 1).^18,19^ Most studies (n = 9, 82%) did not report or explicitly state how missing values were handled, except 1 study which utilized complete case analysis and 1 study that imputed missing values.^15,20^

**Table 1.** Evaluating Performance of Risk Prediction Models

Variable	Parameter	Models, No. (%)
Validation	Retrospective	11 (92)
	Ambispective	1 (8)
	Simulation	1 (8)
	Bootstrapping	3 (25)
Performance metrics	AUROC	12 (100)
	Provided optimal threshold sensitivity and specificity	7 (58)
Calibration	Displayed calibration plot	3 (25)
	Hosmer-Lemeshow statistic	4 (33)

Note. AUROC, area under the receiver operating characteristic.

### Participants

The number of participants in both the model development and validation set(s) were clearly defined for all studies. The median number of participants utilized in model development was 49,231 (IQR, 367–68,809), and the median number of events used to develop models was 303 (IQR, 146–504) (Table 2).

**Table 2.** Counts of Coefficients, Performance, Sample Size, and Events by Study

		Coefficients		Samples			Events (CDI)		
		Candidate	Final	Derivation Set	Total Set	Per Candidate Variable	Derivation Set	Total Set	Per Candidate Variable
Chandra et al	Index model (2012)^16^	10	6	21,541	32,334	3,233.4	121	179	17.9
	External validation only (2014)^15^		6		7,026			62	
Cooper et al (2013)^22^		6	4	29,453	44,236	7,372.7	274	488	81.3
Davis et al (2018)^20^		16	11	75,545	97,130	6,070.6	1,172	1,481	92.6
Garey et al (2008)^21^		19	5	41,224	54,226	2,854.0	288	392	20.6
Press et al (2016)^33^		26	6	40,990	61,482	2,364.7	189	282	10.8
Tabak et al (2015)^19^		30	12		78,080	2,602.7		323	10.8
Oh et al (2018)^18^	MGH model	1,837	20	33,477	65,718	35.8	315	552	0.3
	UM model	4,836	20	155,009	191,014	39.5	1,781	2,141	0.4
Tilton et al	Index model (2019)^14^	15	2		200	13.3		100	6.7
	Validation and model (2021)^13^	22	3		322	14.6		161	7.3
Voicu et al (2021)^17^	Model 1	30	4		367	12.2		25	0.8
	Model 2	30	4		367	12.2		25	0.8

### Outcome

Most studies (n = 10, 91%) identified patients with CDI using either toxin enzyme immunoassays (EIA) targeting glutamate dehydrogenase (GDH) or toxin A/B and/or a nucleic acid amplification test (NAAT) for toxin B gene. Only 2 studies used a 2-step algorithm (EIA and NAAT) to identify patients. One study considered only patients who had a positive cytotoxicity assay (CTA) (Supplementary Table S1 online).^21^ Also, 8 studies (73%) defined HO-CDI as CDI ≥48 hours after hospital admission; 2 studies (18%) defined HO-CDI as CDI ≥72 hours after admission; and 1 (9%) did not provide a clear definition (Table S2).

### Variable selection

**Figure 2.**
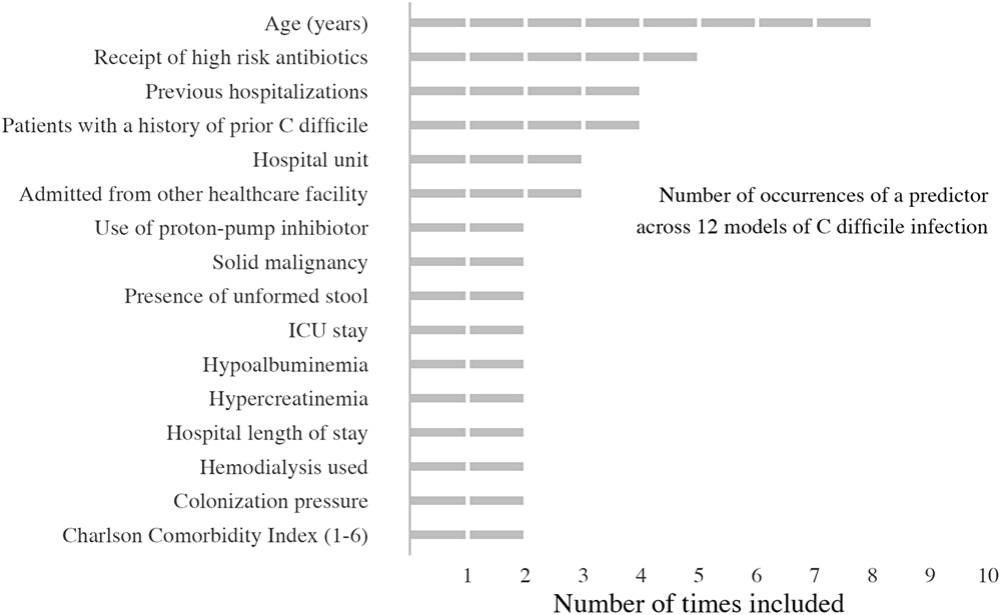
Count of predictors in 12 models of *C. difficile* infection.

**Figure 3.**
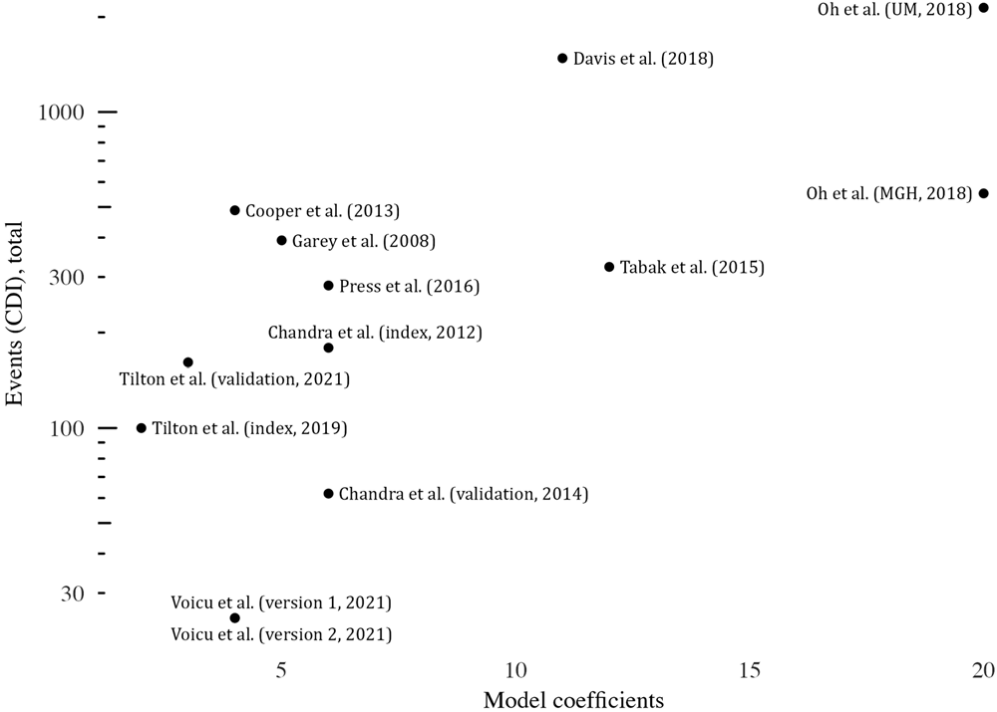
Number of events (CDI infection) versus number of model coefficients.

In total, 6 studies (55%) limited predictors to those present at admission (static), whereas 5 studies (45%) included clinical values that fluctuated throughout hospital admission (dynamic). All but 1 model discretized some or all continuous variables. Most studies (n = 7, 64%) selected variables using stepwise approaches (forward or backward), whereas others used methods that were less susceptible to bias (L2 shrinkage or clustering methods) (Table 3). Models used from 2 to 20 different coefficients in their final model, with a median of 5.5 coefficients (IQR, 4–11.25). The most frequent predictors across all models were age (67%), receipt of ‘high-risk antibiotics’ as defined by literature available at the time of model publication (42%), and a history of either hospitalizations or CDI (each 33%) (Fig. 2 and Supplementary Table S3 online). The median EPV of the studies included in this analysis was 9.1 (IQR, 0.8–18.6), and 4 studies had an EPV <10.^12^ The number of coefficients in each model was modestly correlated with the log of the number of events in the sample size (r = 0.67) (Fig. 3).

**Table 3.** Issues in Model Development

		Treatment of Variables	Missing Data Handled by	Model Building Strategy
Chandra et al	Index model (2012)^16^	Categorized		Stepwise (forward)
	External validation (2014)^15^		Imputed (missing values assumed negative)	
Cooper et al (2013)^22^		No continuous variables	Not specified	Logistic regression (significant correlation to disease state)
Davis et al (2018)^20^		Categorized	Only complete records analyzed	Stepwise (backward)
Garey et al (2008)^21^		Some categorized	Not specified	Stepwise (forward)
Press et al (2016)^33^		Categorized	Not specified	Subsets, minimize χ2 test
Tabak et al (2015)^19^		Categorized	Not specified	Stepwise (backward)
Oh et al (2018)^18^	MGH model	Categorized	Not specified	LASSO (L2)
	UM model	Categorized	Not specified	LASSO (L2)
Tilton et al	Index model (2019)^14^	Categorized	Not specified	Stepwise (backward)
	Validation model (2021)^13^	No continuous variables	Not specified	Stepwise (backward)
Voicu et al (2021)^17^	Model 1	Kept continuous	Not specified	Hierarchical clustering
	Model 2	Kept continuous	Not specified	Hierarchical clustering

### Model validation, performance, and presentation

All studies reported AUROC as a discriminatory measure, with point estimates ranging from 0.68 (fair) to 0.95 (excellent) for final models (median, 0.8; IQR, 0.75–0.80). Most studies (n = 8; 73%) did not report any calibration (agreement between predictions and observations) metrics and 3 studies displayed calibration plots to demonstrate the accuracy of their models over the entire probability range (Table 1).^15,18,19^ Of the 7 models that reported values on validation and derivation sets, 3 models observed a performance penalty between their development and validation performance, and 2 reported surprising improvements in performance when applied to validation data sets (Supplementary Table S4 online). Most studies had a high NPV but a low PPV, with prevalence ranging from 4.1 to 68.1 per 1,000 persons (Supplementary Table S5 online).

### ROB and applicability to review question

**Figure 4.**
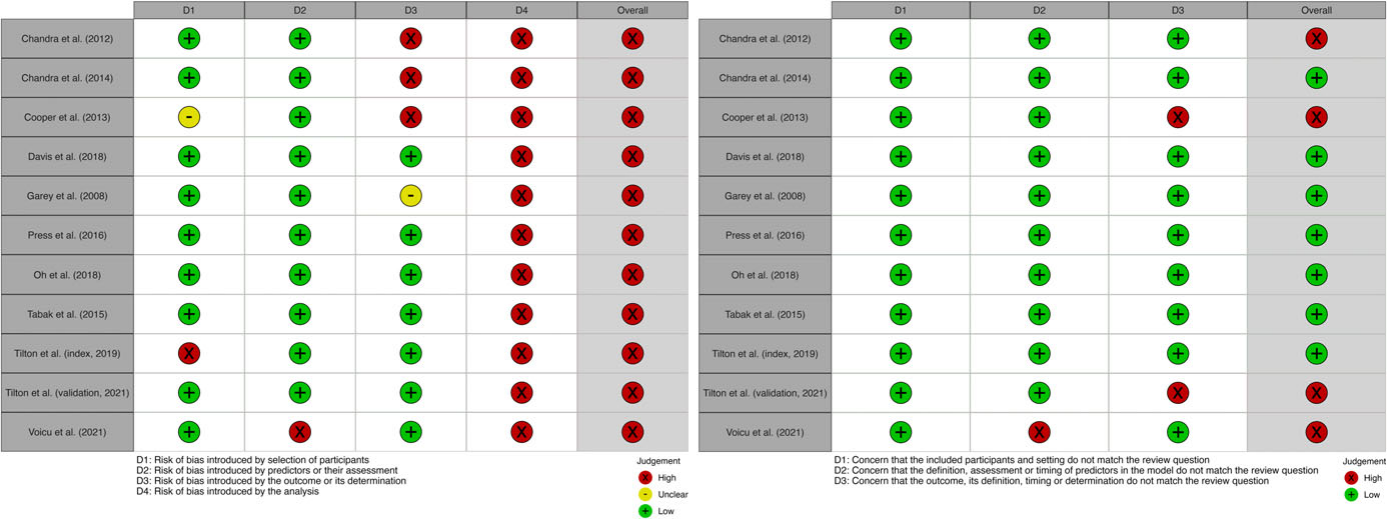
Results from the PROBAST analysis for risk of bias (a) and applicability (b) domain.

Individual domain-specific and overall judgements for the ROB and concerns for applicability are displayed graphically in Figure 4. Here, we briefly summarize the key results from each domain.

- Participants: In the participants domain, almost all studies (n = 9, 82%) had a low ROB. Only 1 study had a high ROB, and the procedure for screening was not specified nor were the exclusion criteria justified in the text.^14^
- Predictors: Most studies (n = 10, 91%) were listed as having a low ROB introduced by predictors or their assessment. One study used variables highly specific to a cirrhosis, which are thus not likely to be externally generalizable.^17^
- Outcome: Many studies (n = 7, 64%) had a low ROB introduced by the outcome or its determination. Three studies had high ROBs due to the inclusion of diarrhea (a predictor in their models) in their outcome definition, and a fourth study changed case definitions over the course of data collection.
- Analysis: All studies were at high risk of bias in the analysis phase. Most studies (n = 7, 64%) used univariable analysis to select the predictors.^13,14,16,17,19,21,22^ All but 2 studies had either unclear or high ROB due to the handling of missing data (either by omitting information or using only complete cases), and almost all did not account for model overfitting and optimism.

All studies had high ROB overall (n = 11, 100%); conversely, most studies had low concern for applicability (n = 7, 64%) (Supplementary Fig. S1 online).

## Discussion

In this systematic review, we assessed the quality and clinical utility of 12 diagnostic prediction models for HO-CDI. Our findings revealed that all studies clearly identified the study design, participant selection process, and outcome definition. Most studies relied on either toxin EIA or NAAT as a standalone test to identify patients with CDI and only 2 studies used a 2-step diagnostic algorithm. The most common predictors across all models included advanced age, receipt of high-risk antibiotics, history of hospitalization, and history of CDI. We identified several methodological issues and reporting deficiencies in the prediction models. Specifically, most studies did not report the occurrence and handling of missing data or model calibration results, and none of them were externally validated. These issues, along with other limitations, contributed to the high ROB and limited reliability and clinical utility of these models.

We evaluated models developed using inpatient data to predict the diagnosis of HO-CDI and assessed their quality and clinical utility. All the reviewed studies exhibited high ROB according to the PROBAST review tool. Notably, only 2 studies explicitly described how they handled missing data,^15,20^ raising concerns about bias and the reliability of conclusions drawn from these data.^23^ One study used complete case analysis without providing a compelling argument for data missing completely at random, which can introduce biased coefficient estimates.^24^ Almost all models discretized some or all continuous predictors, diminishing their predictive power and external validity.^25^

### What is known in the literature and what our study adds

A narrative review published in 2021 discussed some models predicting the risk of incident or recurrent CDI.^26^ The researchers emphasized the need for developing a validated incident CDI model and highlighted the key challenges to model portability. Our report builds on these findings by systematically evaluating and reporting the risk of bias in each study. A systematic review published in 2022 evaluated machine learning models for predicting CDI and outcomes of CDI.^27^ They highlighted that (1) most studies did not use an algorithmic definition of identify CDI electronically and that the definitions were highly variable and (2) none of the studies reported validation of those definitions. Our systematic review is more comprehensive, focusing on all types of diagnostic risk prediction models for HO-CDI, and it underscores the high risk of bias and reporting issues present in these models. We identified 4 broad areas for improvement in future studies.

Our systematic review has identified several methodological issues. Prediction models are prone to overfitting data and their training set or learning spurious correlations.^28^ We evaluated the risk of overfitting by calculating the EPV for each study. The calculated EPV in most studies was <10. However, we may have overestimated this because it was calculated using the number of candidate predictors instead of the number of parameters. A threshold of 10 has been proposed by Peduzzi, but the effect strength and correlation structure may influence this value.^12,29^ Regardless, the median EPV in this analysis was less than the proposed threshold, with notable exceptions of Cooper et al^22^ in 2013 and Davis et al^20^ in 2018 (EPV, 81.3 and 92.6, respectively). Most models in our study used a stepwise approach to variable selection and model calibration, which can introduce bias in the estimation of the regression coefficients.^29,30^ It has been suggested that variable selection should be accompanied by stability investigation, a step not clearly described in any of the studies.^29^

Secondly, all studies exhibited high ROB in the analysis phase of model development, as assessed by PROBAST. Many studies (n = 7, 64%) used a significance threshold (eg, *P* < .05) as a discrimination metric for inclusion in the final prediction model. However, this method can result in errors of inclusion or exclusion; individual predictors are tested for significance only with respect to the outcome, not in the context of information from other predictors.^31,32^

Thirdly, most studies (n = 9, 82%) did not clearly report how they handled missing data.^13–19,22,33^ Excluding missing data without examining the pattern of missingness or using imputation methods can introduce bias in the regression coefficients of prediction models.^34^ Consequently, all studies in our analyses were judged to have an overall high ROB.

Lastly, model performance was difficult to compare because of inconsistent reporting. Although the AUC is the most frequently reported metric to evaluate discrimination, individual performance varies widely. In addition, most models did not report independent derivation and validation sets and lacked calibration metrics. The high negative predictive value (NPV) and low positive predictive value (PPV) observed in most models reflected the low prevalence of CDI in the included studies. These methodological issues raise concerns about the reliability and generalizability of the model’s findings.

Our study had several limitations. We did not include abstracts and conference proceedings in our systematic review because they did not have sufficient data on model development or validation for ROB assessment. It is also possible that some studies were published outside the searched databases, although we attempted to minimize bias by manually searching references and including gray literature. The included studies varied in terms of design, diagnostic tests used to detect CDI, definition of CDI, and analytical methods. In many studies, the data were either insufficient or unavailable for further evaluation.

### Future recommendations

Future studies should consider using a diagnostic algorithm to identify patients with active CDI because it may improve model performance. As recommended by the TRIPOD guidelines, diagnostic models should undergo validation on an external prospective cohort, ensuring their external validity and generalizability.^35^ Lastly, the use of checklists such as PROBAST can help identify sources of bias that often distort model performance.

In conclusion, the diagnostic models for HO-CDI exhibited considerable variation in their ability to estimate the risk of CDI. Most of these models demonstrated methodological shortcomings and provided inadequate information about calibration for feasible implementation in alternative clinical settings. Future studies should consider external validation of their model to improve its generalizability and clinical uptake of their model. A robust model can help identify high-risk patients and enable clinicians to optimize their exposure to high-risk antibiotics during their hospitalization and target them for preventive treatment.

## Supplementary material

For supplementary material accompanying this paper visit https://doi.org/10.1017/ice.2023.185. click here to view supplementary material
