# Supplementary material for: Diagnostic prediction models to identify patients at risk for healthcare-facility–onset Clostridioides difficile: A systematic review of methodology and reporting
Source: Infect Control Hosp Epidemiol. 2023 Sep 4;45(2):174–81. doi: 10.1017/ice.2023.185 (PMC10877537; doi:10.1017/ice.2023.185)
Supplement: Supplementary file 1 [file S0899823X2300185Xsup001.pdf]

Search Strategies for all databases queried

Ovid MEDLINE(R) ALL <1946 to January 20, 2022>

1 Clostridioides difficile/ 10462

2 (clostridioides difficile or clostridium difficile or txid1496 or c diff or cdiff or c difficile or bacillus difficilis or clostridium difficilis or peptoclostridium difficile).mp. 17996

3 1 or 2 17996

4 ((predict* or risk*) and (model* or tool*)).mp. 1237226

5 3 and 4 960

Embase <1974 to 2022 January 20>

1 clostridioides difficile/ 2559

2 (clostridioides difficile or clostridium difficile or txid1496 or c diff or cdiff or c difficile or bacillus difficilis or clostridium difficilis or peptoclostridium difficile).mp. 35578

3 1 or 2 35578

4 ((predict* or risk*) and (model* or tool*)).mp. 1617120

5 3 and 4 1975

Cochrane Database of Systematic Reviews &
Cochrane Central Register of Controlled Trials
January 2022 (77 results)

ID	Search	Hits
#1	MeSH descriptor: [Clostridioides difficile] this term only	207
#2	(("clostridioides difficile" or "clostridium difficile" or "txid1496" or "c diff" or "cdiff" or "c NEXT difficile" or "bacillus difficilis" or "clostridium difficilis" or "peptoclostridium difficile")):ti,ab,kw	1548
#3	#1 or #2	1548
#4	((predict* or risk*) and (model* or tool*)):ti,ab,kw	74874
#5	#3 and #4	77

Table S1 – Test types included in event definition by study.

		**(+) result was included in CDI case definition**		
		**EIA**	**NAAT**	**CTA**
**Chandra et al.**	*Index model (2012)*	Yes	No	No
	External validation (2014)	Yes	No	No
Cooper et al. (2013)		Yes	Yes	No
Davis et al. (2018)		No	Yes	No
Garey et al. (2008)		No	No	Yes
Press et al. (2016)		No	Yes	No
Tabak et al. (2015)		Yes	No	No
**Oh et al. (2018)**	*MGH model*	Yes	*Yes	No
	UM model	Yes	Yes	No
**Tilton et al.**	*Index model (2019)*	No	Yes	No
	Validation model (2021)	No	Yes	No
**Voicu et al. (2021)**	*Model 1*	Yes	No	No
	Model 2	Yes	No	No

*Oh et al only used NAAT for discordant cases between GDH and toxin EIA

Cytotoxicity assay (CTA); enzyme immunoassay (EIA) for toxins; nucleic acid amplification test (NAAT) for toxin gene;

Table S2– Eligibility criteria by study.

		(+) result was included in CDI case definition		
		**Time from admission until considered HO-CDI**	**Criteria to receive laboratory test of CDI**	**Exclusion criteria**
**Chandra et al.**	*Index model (2012)*	≥ 48h	Unformed stool	CDI in last 3 months
	External validation (2014)	≥ 48h		
Cooper et al. (2013)				
Davis et al. (2018)		≥ 48h	Liquid stool	Patients readmitted for primarily for CDI infection
Garey et al. (2008)		≥ 48h	Clinical suspicion	
Press et al. (2016)		≥ 72h	Unformed stool	CDI <3 days, history of CDI
Tabak et al. (2015)		≥ 48h		
**Oh et al. (2018)**	*MGH model*	≥ 48h		Positive in the 14 days prior to admission
	UM model	≥ 48h		Positive in the 14 days prior to admission or admitted to inpatient psychiatric unit.
**Tilton et al.**	*Index model (2019)*	≥ 48h		Prior CDI diagnosis, pregnant, incomplete medical records, IBS, IBD, or diarrhea < 48 hours into admission
	Validation model (2021)	≥ 72h	Receipt of systemic antibiotics	CDI within 90 days prior to admission, pregnant, length of stay < 72 hrs, a diagnosis of IBS or IBD, receipt of metronidazole or vancomycin prior to CDI diagnosis, diarrhea before day 4 of admission
**Voicu et al. (2021)**	*Model 1*	≥ 48h	Diagnosis of variceal bleed secondary to liver cirrhosis	Receipt of metronidazole or vancomycin, recent CDI, CDI treatment prior to testing, antibiotics prior to CDI dignosis, admission from other hospital with different upper GI bleeding protocol
	Model 2	≥ 48h	Diagnosis of variceal bleed	

		(+) result was included in CDI case definition		
		Time from admission until considered HO-CDI	Criteria to receive laboratory test of CDI	Exclusion criteria
			secondary to liver cirrhosis	

Table S3 – List of predictors by study

		Predictors
**Chandra et al.**	*Index model (2012)*	New-onset unformed stool, hospital length of stay >7 days, age >65 years, long-term care facility resident, high-risk antibiotic use, hypoalbuminemia (<30g/L)
	External validation (2014)	Same as index model, see above.
Cooper et al. (2013)		Multiple antibiotics, stool, admission from other facility, prior C. difficile infection
Davis et al. (2018)		Number of high risk antibiotics (0-5), age (<40, 40-55, >55), Charlson comorbidity index (0, 1, >=2), proton pump inhibitor
Garey et al. (2008)		Age (50-80, >80), hemodialysis, non-surgical admission, intensive care unit length of stay
Press et al. (2016)		Age, admission in past 60 days, mechanical vent, dialysis, CHF, antibiotics
Tabak et al. (2015)		CO-CDI pressure >60-percentile, age >64, trasfer from other hospital or skilled nursing facility, mechanical ventilation present on admission, CIM on admission, previous CDI, discharge in last 30 days, admission to intensive care unit, hypoalbuminemia (≤30mg/L), hypercreatininemia (>2 mg/dL), increased bands (>32%), abnormal platelets (<=150k or >420k), and leukocytosis (> 11k).
**Oh et al. (2018)**	*MGH*	Risk factors: Medicine service, CDI in the prior year, propofol, Age 77-89, Chlorhexidine, MICU, ceruloplasmin (.0126-.0214), prescribed metronidazole, prescribed dextrose, prescribed cefepime. Protective factors: OBGYN service, ceruloplasmin (< .001), Surgery service, age (41-56), prescribed simvastatin, prescribed oxycodone, Obstetrics service, Age (18-41), prescribed docusate, admitted from ED.

		Predictors
	UM	Risk factors: CDI in prior year, ED location, tachycardia, prescribed cefoxitin, prescribed fluconazole, prescribed ondansetron, prescribed prochlorperazine, prescribed an antifungal, prescribed an antiemetic, admit hold. Protective factors: prescribed warfarin, age (35-51), prescribed NSAIs, prescribed hydrocodone, on the neurology unit, on the orthopedic surgery unit, on the OB unit, prescribed ibuprofen, living in Washtenaw County, age (18-35).
**Tilton et al.**	*Index model (2019)*	Age (>70), hospitalization (past 90 days)
	Validation model (2021)	History of hematologic mallignancy, history of solid tumor malignancy, hospitalization (past 90 days)
**Voicu et al. (2021)**	*Model 1*	Age, days of admission, Charlson comorbidity index, Child-Pugh score
	Model 2	History of hepatocellular carcinoma, prescribed proton-pump inhibitor, creatinine, urea

Oh et al. used an EMR based ML model and the full list of predictors includes over 1000 variables. Predictors presented reflect the top 10 positive and negative predictors in the model.

Table S4 - Model performance on derivation and validation sets.

		Performance	
		**Derivation**	**Validation**
**Chandra et al.**	*Index model (2012)*	0.93 (95% CI: 0.92, 0.95)	0.95 (95% CI: 0.93, 0.96)
	External validation (2014)		0.94 (95% CI: 0.93, 0.96)
Cooper et al. (2013)		0.93 (95% CI: 0.93, 0.93)	0.91 (95% CI: 0.90, 0.91)
Davis et al. (2018)		0.75 (95% CI: NR)	0.77 (95% CI: NR)
Garey et al. (2008)		0.73 (95% CI: NR)	0.68 (95% CI: NR)
Press et al. (2016)		0.85 (95% CI: NR)	0.85 (95% CI: NR)
Tabak et al. (2015)		0.79 (95% CI: 0.76, 0.81)	0.79 (95% CI: 0.76, 0.81)
**Oh et al. (2018)**	*MGH model*		0.75 (95% CI: 0.73, 0.78)
	UM model		0.82 (95% CI: 0.8, 0.84)
**Tilton et al.**	*Index model (2019)*		0.7 (95% CI: NR)
	Validation model (2021)		0.74 (95% CI: NR)
**Voicu et al. (2021)**	*Model 1*		0.84 (95% CI: 0.76, 0.92)
	Model 2		0.82 (95% CI: 0.75, 0.90)

Value presented in Tilton et al. 2021 is for the new model developed in that paper. The validation performance of the index model in the 2021 Tilton paper is .62.

Table S5 - Model performance on derivation and validation sets.

Paper	Model	Estimated study prevalence (per 1000 persons)	Sensitivity	Specificity	PPV	NPV	LR+	LR-
**Chandra et al.**	*Index model (2012)*	5.5	0.94	0.80	0.03	1.00	4.70	0.08
	External validation only (2014)	8.8	0.98	0.85	0.06	1.00	6.64	0.02
Cooper et al. (2013)		11	0.92	0.87	0.07	1.00	7.03	0.10
Davis et al. (2018)		15.2	0.82	0.52	0.03	0.99	1.73	0.34
Garey et al. (2008)		7.2	*NA*	*NA*	*NA*	*NA*	*NA*	*NA*
Press et al. (2016)		4.6	0.82	0.76	0.02	1.00	3.37	0.24
Tabak et al. (2015)*		4.1	0.75	0.71	0.01	1.00	2.59	0.35
**Oh et al. (2018)**	*MGH model*	8.4	0.23	0.95	0.04	0.99	4.83	0.81
	UM model	11.2	0.28	0.95	0.06	0.99	5.79	0.76
**Tilton et al.**	*Index model (2018)*	*NA*	0.44	0.80	*NA*	*NA*	2.20	0.70
	Validation & model (2021)	*NA*	0.63	0.78	*NA*	*NA*	2.42	0.67
**Voicu et al. (2021)***	*Model 1*	68.1	0.68	0.88	0.29	0.97	5.67	0.36
	Model 2	68.1	0.68	0.74	0.16	0.97	2.62	0.43

Study prevalence calculated from population information provided in manuscript. Tilton et al. only provided information on their case-control sample.
*Sensitivity and specificity estimated from ROC figure.

Figure S1

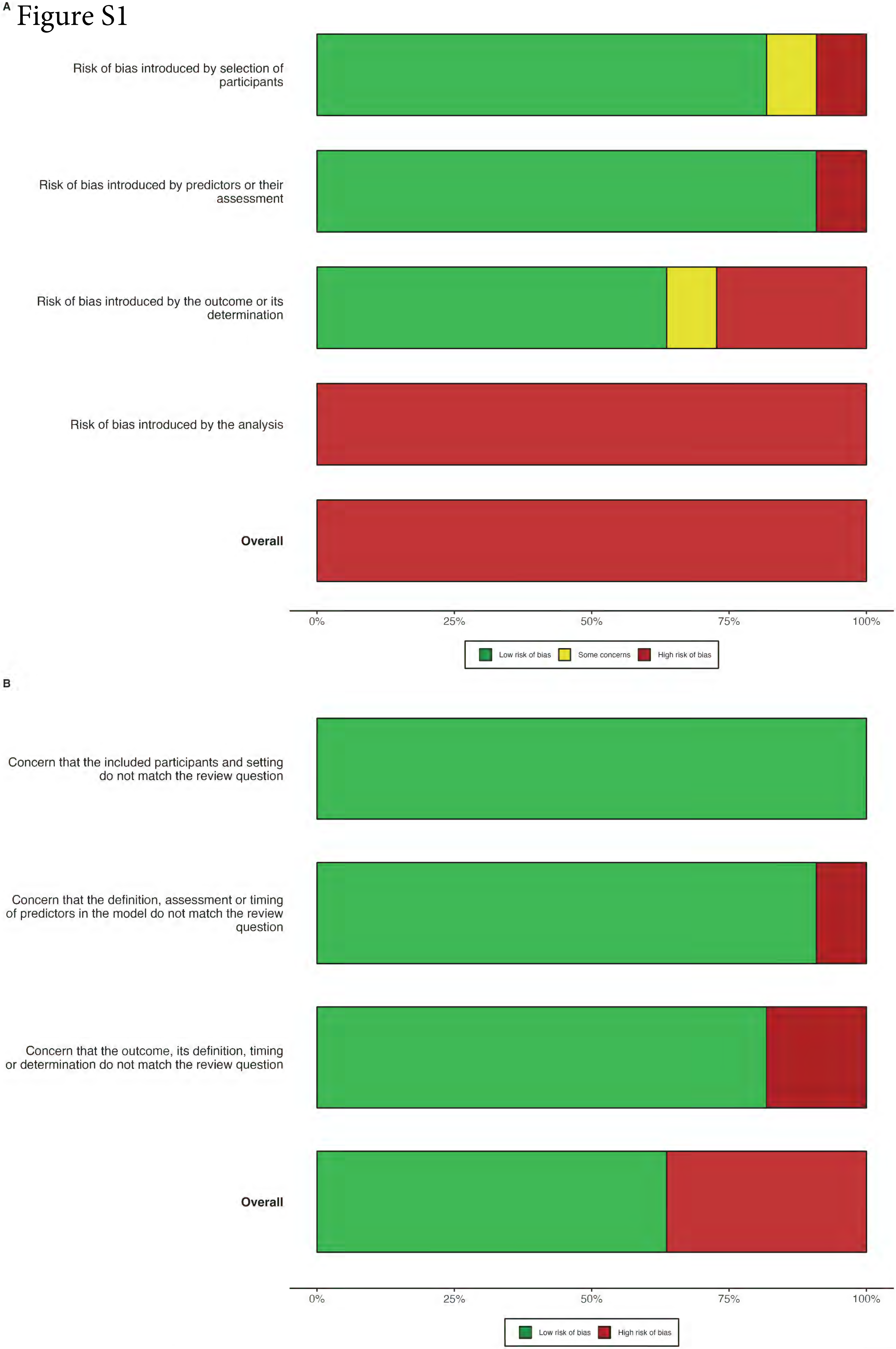
A
Risk of bias introduced by selection of participants
Risk of bias introduced by predictors or their assessment
Risk of bias introduced by the outcome or its determination
Risk of bias introduced by the analysis
Overall
0%
25%
50%
75%
100%
Low risk of bias
Some concerns
High risk of bias
B
Concern that the included participants and setting do not match the review question
Concern that the definition, assessment or timing of predictors in the model do not match the review question
Concern that the outcome, its definition, timing or determination do not match the review question
Overall
0%
25%
50%
75%
100%
Low risk of bias
High risk of bias

PRISMA 2020 Checklist

Section and Topic	Item #	Checklist item	Location where item is reported
**TITLE**			
Title	1	Identify the report as a systematic review.	1
**ABSTRACT**			
Abstract	2	See the PRISMA 2020 for Abstracts checklist.	
**INTRODUCTION**			
Rationale	3	Describe the rationale for the review in the context of existing knowledge.	3
Objectives	4	Provide an explicit statement of the objective(s) or question(s) the review addresses.	3-4
**METHODS**			
Eligibility criteria	5	Specify the inclusion and exclusion criteria for the review and how studies were grouped for the syntheses.	4-5
Information sources	6	Specify all databases, registers, websites, organisations, reference lists and other sources searched or consulted to identify studies. Specify the date when each source was last searched or consulted.	4
Search strategy	7	Present the full search strategies for all databases, registers and websites, including any filters and limits used.	4-5, supplement
Selection process	8	Specify the methods used to decide whether a study met the inclusion criteria of the review, including how many reviewers screened each record and each report retrieved, whether they worked independently, and if applicable, details of automation tools used in the process.	5
Data collection process	9	Specify the methods used to collect data from reports, including how many reviewers collected data from each report, whether they worked independently, any processes for obtaining or confirming data from study investigators, and if applicable, details of automation tools used in the process.	5-6
Data items	10a	List and define all outcomes for which data were sought. Specify whether all results that were compatible with each outcome domain in each study were sought (e.g. for all measures, time points, analyses), and if not, the methods used to decide which results to collect.	5
	10b	List and define all other variables for which data were sought (e.g. participant and intervention characteristics, funding sources). Describe any assumptions made about any missing or unclear information.	5
Study risk of bias assessment	11	Specify the methods used to assess risk of bias in the included studies, including details of the tool(s) used, how many reviewers assessed each study and whether they worked independently, and if applicable, details of automation tools used in the process.	6
Effect measures	12	Specify for each outcome the effect measure(s) (e.g. risk ratio, mean difference) used in the synthesis or presentation of results.	NA
Synthesis methods	13a	Describe the processes used to decide which studies were eligible for each synthesis (e.g. tabulating the study intervention characteristics and comparing against the planned groups for each synthesis (item #5)).	NA
	13b	Describe any methods required to prepare the data for presentation or synthesis, such as handling of missing summary statistics, or data conversions.	NA
	13c	Describe any methods used to tabulate or visually display results of individual studies and syntheses.	NA
	13d	Describe any methods used to synthesize results and provide a rationale for the choice(s). If meta-analysis was performed, describe the model(s), method(s) to identify the presence and extent of statistical heterogeneity, and software package(s) used.	NA
	13e	Describe any methods used to explore possible causes of heterogeneity among study results (e.g. subgroup analysis, meta-regression).	NA
	13f	Describe any sensitivity analyses conducted to assess robustness of the synthesized results.	NA
Reporting bias assessment	14	Describe any methods used to assess risk of bias due to missing results in a synthesis (arising from reporting biases).	6
Certainty assessment	15	Describe any methods used to assess certainty (or confidence) in the body of evidence for an outcome.	NA

PRISMA 2020 Checklist

Section and Topic	Item #	Checklist item	Location where item is reported
**RESULTS**			
Study selection	16a	Describe the results of the search and selection process, from the number of records identified in the search to the number of studies included in the review, ideally using a flow diagram.	7
	16b	Cite studies that might appear to meet the inclusion criteria, but which were excluded, and explain why they were excluded.	7
Study characteristics	17	Cite each included study and present its characteristics.	7
Risk of bias in studies	18	Present assessments of risk of bias for each included study.	10
Results of individual studies	19	For all outcomes, present, for each study: (a) summary statistics for each group (where appropriate) and (b) an effect estimate and its precision (e.g. confidence/credible interval), ideally using structured tables or plots.	7-8
Results of syntheses	20a	For each synthesis, briefly summarise the characteristics and risk of bias among contributing studies.	NA
	20b	Present results of all statistical syntheses conducted. If meta-analysis was done, present for each the summary estimate and its precision (e.g. confidence/credible interval) and measures of statistical heterogeneity. If comparing groups, describe the direction of the effect.	NA
	20c	Present results of all investigations of possible causes of heterogeneity among study results.	NA
	20d	Present results of all sensitivity analyses conducted to assess the robustness of the synthesized results.	NA
Reporting biases	21	Present assessments of risk of bias due to missing results (arising from reporting biases) for each synthesis assessed.	10
Certainty of evidence	22	Present assessments of certainty (or confidence) in the body of evidence for each outcome assessed.	NA
**DISCUSSION**			
Discussion	23a	Provide a general interpretation of the results in the context of other evidence.	11
	23b	Discuss any limitations of the evidence included in the review.	13-14
	23c	Discuss any limitations of the review processes used.	13-14
	23d	Discuss implications of the results for practice, policy, and future research.	14
**OTHER INFORMATION**			
Registration and protocol	24a	Provide registration information for the review, including register name and registration number, or state that the review was not registered.	Not registered
	24b	Indicate where the review protocol can be accessed, or state that a protocol was not prepared.	NA
	24c	Describe and explain any amendments to information provided at registration or in the protocol.	NA
Support	25	Describe sources of financial or non-financial support for the review, and the role of the funders or sponsors in the review.	15
Competing interests	26	Declare any competing interests of review authors.	15
Availability of data, code and other materials	27	Report which of the following are publicly available and where they can be found: template data collection forms; data extracted from included studies; data used for all analyses; analytic code; any other materials used in the review.	15

From: Page MJ, McKenzie JE, Bossuyt PM, Boutron I, Hoffmann TC, Mulrow CD, et al. The PRISMA 2020 statement: an updated guideline for reporting systematic reviews. BMJ 2021;372:n71. doi: 10.1136/bmj.n71

TRIPOD-SRMA Checklist for reporting systematic reviews of prediction model studies

Section and topic	Item No	Checklist item	Page
**Title**			
Title	1	Identify the report as a systematic review or meta-analysis (or both) of diagnostic or prognostic model studies. Specify the target population and outcome(s) predicted as relevant to the review question.	1
**Abstract**			
Abstract	2	See the TRIPOD-SRMA Checklist for Abstracts	1
**Introduction**			
Rationale	3	Describe the rationale for the review in the context of existing knowledge.	3
Objectives	4	Provide an explicit statement of the objective(s) being addressed with reference to: target population, index and comparator models (as relevant), outcome(s), time (prediction horizon and intended moment of using the model), and setting.	3
**Methods**			
Study eligibility criteria	5	Specify study characteristics used as eligibility criteria, including any prediction models of specific interest, and whether development or validation studies (or both) were eligible.	4
Information sources	6	Specify all databases, registers, websites, organisations, reference lists and other sources searched or consulted to identify studies. Specify the date when each source was last searched or consulted.	4
Search strategy	7	Present the full search strategies for all databases, registers and websites, including any filters and limits used.	Supplement
Study selection process	8	Specify the methods used to decide whether a study met the inclusion criteria of the review, including how many reviewers screened each record and each report retrieved, whether they worked independently, and if applicable, details of automation tools used in the process.	4-5
Data collection process	9	Specify the methods used to collect data from study reports, including how many reviewers collected data from each report, whether they worked independently, any processes for obtaining or confirming data from study investigators, and if applicable, details of automation tools used in the process.	5-6
Data Items	10a	List and define all items for which data were sought from each study.	5
	10b	State the model performance measures that were sought (e.g., measures of calibration, discrimination, overall model fit, clinical utility).	5-6
	10c	Describe how any desired but unreported data items (items 10a, 10b) were handled (e.g., contacted authors, calculated from other reported information).	5-6
Risk of bias and applicability assessment	11	Specify the methods used to assess risk of bias in the included studies and their applicability to the review question. This should be done separately for each model development and validation. Include details of any tool(s) used, how many reviewers assessed each study and whether they worked independently.	6
Synthesis methods	12a	Describe any methods for synthesising estimates of performance measures for each model. If meta-analysis was carried out, describe the methods used, including any transformations of data prior to pooling, how any heterogeneity in model performance was quantified and handled, and software package(s) used.	NA
	12b	Describe any methods used to explore possible causes of heterogeneity in model performance (e.g., subgroup analysis, meta-regression), including whether or not they were planned.	NA

Section and topic	Item No	Checklist item	Page
	12c	Describe any sensitivity analyses conducted to assess robustness of the synthesised results.	NA
Certainty assessment	13	Describe any methods used to assess certainty (or confidence) in the body of evidence for a prediction model.	NA
**Results**			
Study selection	14	Describe the results of the search and selection process, from the number of records identified in the search to the number of studies and models included in the review, ideally using a flow diagram.	7
Study and model characteristics	15	Present study characteristics and model details extracted (as per Item 10a), and cite the study reports.	7-9
Risk of bias and applicability	16	Present results of risk of bias and applicability assessment. This should be done separately for each model development and validation in each included study.	10
Results of model performance in individual studies	17	Present performance estimates and confidence intervals for each model and all evaluations, including whether they relate to the internal or external validation performance. If internal, give details of the method.	9
Results of syntheses	18a	Present the results of any synthesis of model performance, together with details of which study estimates contributed. If meta-analysis was carried out, then for each model and performance measure, present summary results, confidence/credible intervals and measures of heterogeneity. Forest plots may be useful.	NA
	18b	For each model, present results of all investigations of possible causes of heterogeneity in model performance.	NA
	18c	Present results of all sensitivity analyses conducted to assess the robustness of the synthesised results.	NA
Certainty of evidence	19	Present any assessments of certainty (or confidence) in the body of evidence for each prediction model of interest.	NA
**Discussion**			
Summary of evidence	20	Summarise the main findings including the strengths and limitations of the evidence.	11
Limitations	21	Discuss the strengths and limitations of the review process.	13-14
Implications	22	Discuss implications of the results in the context of other evidence and for practice, policy, and future research.	11-13
**Other information**			
Registration and protocol	23a	Provide registration information for the review, including register name and registration number, or state that the review was not registered.	Not registered
	23b	Indicate where the review protocol can be accessed, or state that a protocol was not prepared.	NA
	23c	Describe and explain any amendments to information provided at registration or in the protocol.	NA
Support	24	Describe sources of financial or non-financial support for the review, and the role of the funders or sponsors in the review.	15
Competing interests	25	Declare any competing interests of review authors.	15

Section and topic	Item No	Checklist item	Page
Availability of data, code, and other materials	26	Report which of the following are publicly available and where they can be found: template data collection forms; data extracted from included studies; data used for all analyses; analytic code; any other materials used in the review.	15

This checklist appears in appendix 2 of Snell KIE, Levis B, Damen JAA, et al. Transparent reporting of multivariable prediction models for individual prognosis or diagnosis: checklist for systematic reviews and meta-analyses (TRIPOD-SRMA). *BMJ* 2023;381:e073538. doi:10.1136/bmj-2022-073538.
